# Comparative analysis of the image quality and diagnostic performance of the zooming technique with diffusion-weighted imaging using different b-values for thyroid papillary carcinomas and benign nodules

**DOI:** 10.3389/fonc.2024.1241776

**Published:** 2024-05-07

**Authors:** Liling Jiang, Jiao Chen, Yong Tan, Jian Wu, Junbin Zhang, Daihong Liu, Jiuquan Zhang

**Affiliations:** ^1^ Department of Radiology, Shapingba Hospital affiliated to Chongqing University (Shapingba District People’s Hospital of Chongqing), Chongqing, China; ^2^ Department of Radiology, Chongqing University Cancer Hospital, Chongqing, China; ^3^ Head and Neck Cancer Center, Chongqing University Cancer Hospital, Chongqing, China

**Keywords:** diffusion-weighted imaging, b-value, thyroid nodule, image quality, diagnostic performance

## Abstract

**Objective:**

To compare image quality and diagnostic performance using different b-values for the zooming technique with diffusion-weighted imaging (ZOOMit-DWI) in thyroid nodules

**Materials and methods:**

A total of 51 benign thyroid nodules and 50 thyroid papillary carcinomas were included. ZOOMit-DWI was performed with b-values of 0, 500, 1000, 1500 and 2000 s/mm^2^. The sharpness was evaluated as subjective index. The signal intensity ratio (SIR), signal-to-noise ratio (SNR) and apparent diffusion coefficient (ADC) were measured as objective indices. Pairwise comparisons were performed among the different b-value groups using the *Friedman* test. A receiver operating characteristic curve of the ADC value was used to evaluate diagnostic performance. The DeLong test was used to compare diagnostic effectiveness among the different b-value groups

**Results:**

In both the papillary carcinoma group (*P* = 0.670) and the benign nodule group (*P* = 0.185), the sharpness of nodules was similar between b-values of 1000 s/mm^2^and 1500 s/mm^2^. In the papillary carcinoma group, the SIR_nodule_ was statistically higher in DWI images with a b-value of 1500 s/mm^2^than in DWI images with b-values of 500 s/mm^2^(*P* = 0.004), 1000 s/mm^2^(*P* = 0.002), and 2000 s/mm^2^(*P* = 0.003). When the b-values were 1500 s/mm^2^(*P* = 0.008) and 2000 s/mm^2^(*P* = 0.009), the SIR_nodule_ significantly differed between the papillary carcinoma group and the benign nodule group. When b = 500 s/mm^2^, the ADC had an AUC of 0.888. When b = 1000 s/mm^2^, the ADC had an AUC of 0.881. When b = 1500 s/mm^2^, the ADC had an AUC of 0.896. When b = 2000 s/mm^2^, the ADC had an AUC of 0.871. The DeLong test showed comparable diagnostic effectiveness among the different b-value groups except for between b-values of 2000 s/mm^2^and 1500 s/mm^2^, with a b-value of 2000 s/mm^2^showing lower effectiveness

**Conclusion:**

This study suggests that 1500 s/mm^2^may be a suitable b-value to differentiate benign and malignant thyroid nodules in ZOOMit-DWI images, which yielded better image quality

## Introduction

Thyroid nodules are found in 19-67% of asymptomatic individuals using ultrasonography (1). Among these nodules, 10-15% of nodules are malignant (2). In patients with malignant nodules, early diagnosis and active follow-up treatment can elevate the 10-year survival rate to 90% (3). Therefore, it is essential to find a reliable non-invasive imaging tool to diagnose malignant thyroid nodules.

Ultrasound is a noninvasive technique for identifying thyroid nodules; however, the result can vary depending on the evaluator (4). Fine needle aspiration is an invasive examination, and one-third of the results are inconclusive (2). Computed tomography is limited in differentiating between malignant and benign thyroid nodules and has the disadvantage of radiation exposure. Many authors have investigated the important value of diffusion-weighted imaging (DWI) in differentiation between malignant and benign thyroid nodules (3, 5–8). With the development of magnetic resonance imaging (MRI), DWI has become a popular modality for identifying thyroid nodules in recent years and can assess the Brownian motion of water molecules at the cellular level (9).

In malignant nodules, water movement is restricted due to increased cellularity and reduced extracellular space. In most previous thyroid studies, the b-value of DWI was lower than 1000 s/mm^2^ for differentiation between benign and malignant lesions (3, 5–8). With the advancement of high-field and DWI MRI techniques, high-b-value DWI of the thyroid has become much simpler. Past studies have shown that a high b-value of 2000 s/mm^2^ is optimal for the diagnosis and differential diagnosis of thyroid nodules (10, 11). However, only a b-value of 800 s/mm^2^was compared to 2000 s/mm^2^ in the above study, and the diagnostic performance of b-values from 800 to 2000 s/mm^2^ is unknown.

In MRI exams, the b-value is an index indicating the degree of sensitivity to diffusion in the images. The choice of b-value does, to some extent affect the distortion as the eddy currents may be weaker for smaller b-values, but susceptibility arguably is the main source of image distortion in DWI. Higher b values produce increased signal attenuation and usually requires increased signal averaging to compensate for the signal-to-noise ratio. Most malignant thyroid nodules were papillary carcinomas. The community still have not reached a consensus regarding the optimal b value for MRI exams to detect thyroid papillary carcinomas. Therefore, the optimal b-value to differentiate between thyroid papillary carcinomas and thyroid benign nodules in clinical applications must be explored.

In this study, we combined image quality and differential diagnostic performance to determine the optimal b-value for DWI detection of thyroid papillary carcinomas and thyroid benign nodules. Image quality included subjective and objective aspects. The zooming technique with diffusion-weighted imaging (ZOOMit-DWI) was used in this study, which entails a reduced field of view. We hypothesized that ZOOMit-DWI would show excellent performance in the thyroid. We aimed to identify the optimal b-value for differentiating thyroid papillary carcinomas and thyroid benign nodules.

## Materials and methods

### Patient enrollment and thyroid nodule selection

All procedures performed in this study involving human participants were in accordance with the ethical standards of the research committee and approved by the local research committee (IRB No. CZLS2021207-A). Informed consent was signed by all study participants. The study recruited 95 consecutive patients in Chongqing University Cancer Hospital from July 2021 to May 2022. All patients underwent thyroid MRI examinations in this study. Data collection was planned before surgical pathological results were performed.

The inclusion criteria were as follows: a) planned thyroid nodule surgical treatment; b) no needle biopsy or treatment before surgery; c) the pathologic finding was thyroid papillary carcinoma or thyroid benign nodule. The exclusion criteria were as follows: a) contraindications to MRI examination; b) obvious artifacts on DWI; c) incomplete DWI imaging data; d) the pathologic finding was borderline neoplasm.

### Sample size

There are no generally accepted approaches to estimate the sample size requirements for derivation studies, however, we ensured that the study met suggested requirements of having at least 10 events per candidate variable for the derivation of a model.

### Examination method

In this study, Prisma 3.0 T MRI (Simens Healthcare, Germany Erlangen) was used for examination on MRI with a 16­channel surface coil (Zhongzhi Medical, China Jiangsu). ZOOMit-DWI was performed with the following diffusion gradient b factors: 0, 500, 1000, 1500 and 2000 s/mm^2^. ZOOMit-DWI uses the availability of fully independent parallel radiofrequency transmission coils, allowing excitation of selective “inner volumes” (12). When the field of view is reduced, the readout tends to be faster which decreases the susceptibility artifacts. The smaller field of view can also be used to enable higher spatial resolution without increasing the readout duration (13). The imaging parameters for DWI were TR: 4600 ms; TE: 72 ms; FOV (RL x AP): 160 × 58 mm; average: 1 (b=0 s/mm^2^), 4 (b=500 s/mm^2^), 6 (b=1000 s/mm^2^), 9 (b=1500 s/mm^2^), and 13(b=2000 s/mm^2^); matrix size (RL x AP): 110 × 36 mm; slice thickness: 3 mm; intersection gap: 0.3 mm; diffusion gradient orientations: 3; flip angle:150; and examination time: 439 s. The imaging parameters for T2WI were TR: 3000 ms; TE: 88 ms; FOV: 200 × 200mm; average: 4; matrix size: 256×256; slice thickness: 3 mm; intersection gap: 0.3 mm; flip angle:150; and examination time: 158 s.

### Image analysis

All morphological images and DWI images were evaluated in Siemens workstation (syngo.via). DWI images were evaluated by a radiologist (LLJ). To evaluate reproducibility, all nodules were evaluated one month later by the same radiologist (LLJ) and one other radiologist (JC). Image quality analysis included subjective and objective aspects. Diagnostic efficacy was evaluated according to differential diagnostic performance. Only the largest nodule was evaluated in one lobule.

#### Subjective image quality analysis

Subjective image quality of nodules and thyroids on DWI images with different b-values was evaluated respectively according to 4-point scale depending on sharpness: 4 = the boundary was clearly depicted; 3 = the boundary was unclearly depicted; 2 = the boundary was indistinctly visible; 1 = the nodule or thyroid cannot be displayed.

#### Objective image quality analysis

Signal intensity (SI) and standard deviation (SD) was measured in the nodule, thyroid and air in the same slice. The region of interest (ROI) of nodules was manually drawn along the nodule margin. The ROI of the thyroid was the largest and usually circularly drawn on normal regions of the thyroid gland. The ROI for air was circularly drawn around air in the trachea. First, an ROI was drawn on DWI image with b = 0 s/mm^2^. Then, the ROI was copied to DWI images with b = 500, 1000, 1500, and 2000 s/mm^2^.The cystic and hemorrhagic areas were avoided. Then, the signal intensity ratio (SIR) of nodules and signal-to-noise ratio (SNR) of thyroids were calculated according to the following formula: SIR_nodule_=(SI_nodule_−SI_thyroid_)/SI_thyroid_; SNR_thyroid_=SI_thyroid_/SD_air_ (14).

#### Differential diagnostic efficacy analysis

The mean apparent diffusion coefficient (ADC) was measured in different ADC maps (b = 500, 1000, 1500, 2000 s/mm^2^). ADC calculated using the following formulas: ADC=In(S0/S1)/(b1-b0). The b0 mean 0. The b1 mean 500, 1000, 1500 0r 2000. S0 represented the signal intensity of ROI in the DWI images of b = 0 s/mm^2^. S1 represented the signal intensity of ROI in the DWI images of b = 500, 1000, 1500 0r 2000 s/mm^2^. The extraction of the ADC values was automatically performed by the Siemens workstation (syngo.via).

Four ADC values were recorded for each nodule. An ROI was manually drawn along the nodule margin. First, the ROI was drawn on a DWI image with b = 0 s/mm^2^, and then the ROI was copied to different ADC maps. The above ROI was in the same slice, and cystic and hemorrhagic areas were avoided.

### Pathology

According to long-time clinic experience, the histopathologic examination was used as the gold standards. All surgically resected nodules were subjected to an intra-operative frozen section for preliminary risk assessment. If the nodule was diagnosed as a benignancy by intra-operative frozen section, total thyroidectomy would not be applied and the specimen of lobectomy would be acquired for further paraffin section. If the nodule was suspicious malignant or atypical, total thyroidectomy would be further given and the specimen of total thyroidectomy would be acquired for further paraffin section and immunohistochemical staining. According to pathological examination results, the nodules were assigned to either the papillary carcinoma group or benign nodule group.

### Statistical analysis

The statistical analyses were calculated on personal computers utilizing the Statistical Package for Social Sciences (SPSS−windows version 25.0). All variabilities of subjective and objective evaluation derived from MRI were exploratory. The interobserver and intraobserver variability were assessed by determining intraclass correlation coefficients, with the values of <0.50, 0.50–0.75, 0.75–0.90, >0.90 reflecting poor, moderate, good and excellent correlations (15). The interobserver agreement selected two-way random model and intraobserver agreement selected two-way mixed model. Subjective and objective evaluation results were compared among the different b-value groups. The Kolmogorov–Smirnov test was performed to analyze normality. According to the results of the Kolmogorov–Smirnov test, Student’s *t* test or Mann-Whitney *u* test was used to compare difference between papillary carcinomas and benign nodules. The data did not conform to a normal distribution. *Friedman* test was applied to assess whether significant differences existed among different b-value groups. Bonferroni correction was a useful technique for controlling the family-wise error rate in multiple comparisons. The area under the receiver operating characteristic (ROC) curve was also calculated. From ROC curve analysis on different b values, an optimal cut-off value of ADC to predict thyroid papillary carcinoma was determined by using Youden index. Youden’s index = sensitivity + specificity−1. Based on this data-driven cut-off value, sensitivity, specificity and 95% confidence intervals were calculated. The DeLong test was used to assess the area under the curve values and compare diagnostic effectiveness among the different b-value groups.

## Results

### Clinical data

**Figure 1:**
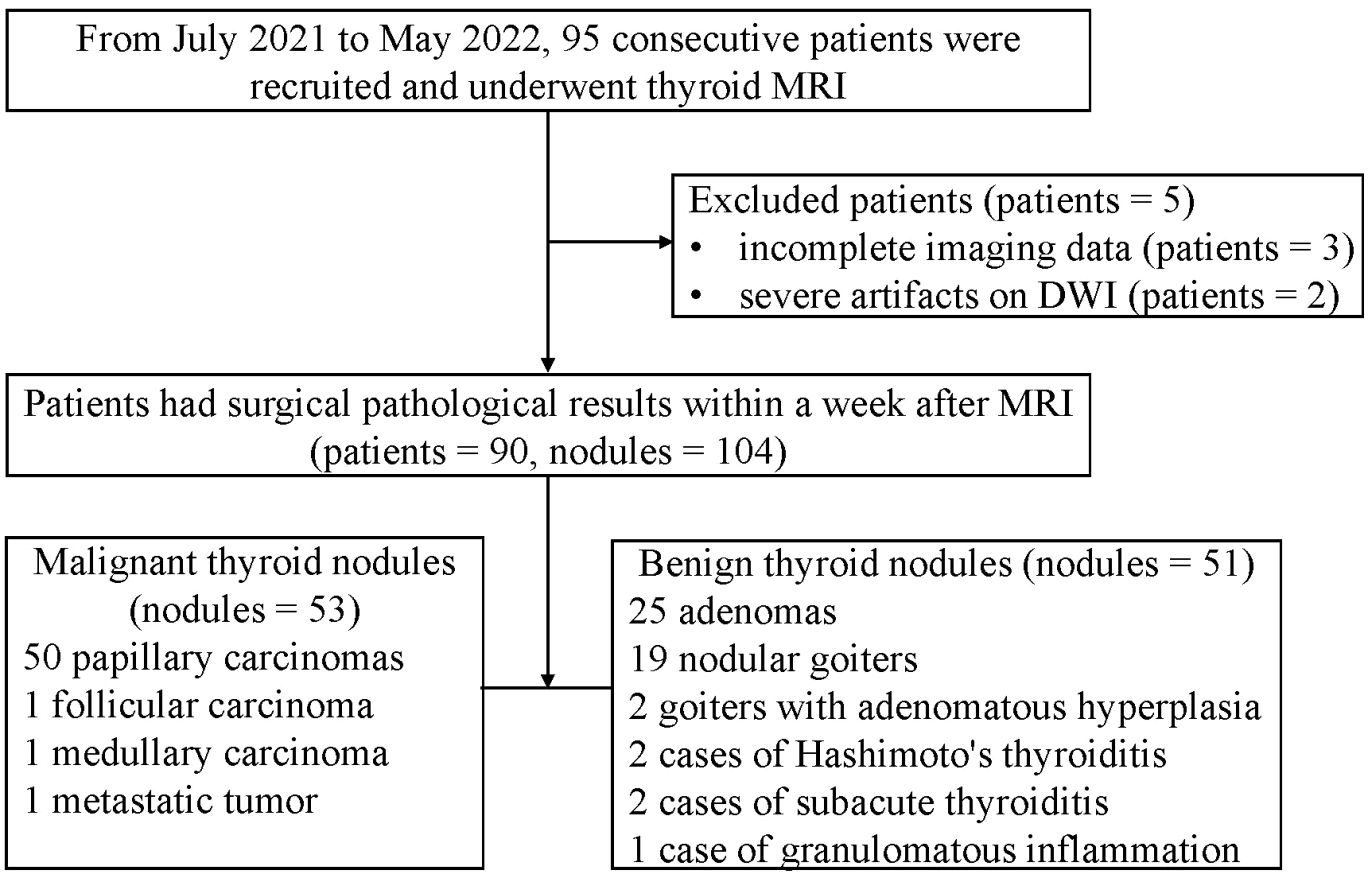
Flowchart of this study.

Of the 95 patients, 5 patients were excluded (3 with incomplete imaging data, 2 with severe artifacts on DWI, 4 were females, 1 was male, with ages ranging from 43 to 61 years old). Ultimately, the 104 nodules from 90 patients were included in this study. 68 were females, and 22 were males, and ages were 47.69 ± 12.09 ranging from 21 to 77 years old. Patients had surgical pathological results within a week after MRI. 53 nodules were malignant (50 papillary carcinomas, 1 follicular carcinoma, 1 medullary carcinoma, 1 metastatic tumor), and 51 nodules were benign (25 adenomas, 19 nodular goiters, 2 goiters with adenomatous hyperplasia, 2 cases of Hashimoto’s thyroiditis, 2 cases of subacute thyroiditis, 1 case of granulomatous inflammation). There was not any adverse event in this study. The flow chart was showed in **Figure 1**.

### Interobserver and intraobserver agreement

Interobserver agreement was moderate and good (0.746-0.965) for subjective evaluations of thyroids and nodules, respectively (**Table 1**). Interobserver agreement was moderate and good (0.739-0.979) for objective evaluations of thyroids and nodules (**Table 1**). Intraobserver agreement was good and excellent (0.908-0.978) for subjective evaluations of thyroids and nodules, respectively (**Table 1**). Intraobserver agreement was good and excellent (0.811-0.995) for objective evaluations of thyroids and nodules (**Table 1**).

**Table 1:** The interobserver and intraobserver agreement of measurements of thyroid nodules.

		Inter-observer		Intrao-bserver	
		ICC value	95% CI	ICC value	95% CI
Signal intensity	Nodule (b=500)	0.929	0.846-0.985	0.995	0.992-0.997
	Thyroid (b=500)	0.834	0.706-0.921	0.991	0.987-0.994
	Air (b=500)	0.892	0.840-0.927	0.992	0.988-0.994
	Nodule (b=1000)	0.898	0.760-0.899	0.991	0.988-0.995
	Thyroid (b=1000)	0.889	0.839-0.890	0.992	0.988-0.995
	Air (b=1000)	0.862	0.796-0.907	0.992	0.989-0.995
	Nodule (b=1500)	0.953	0.792-0.991	0.993	0.990-0.996
	Thyroid (b=1500)	0.829	0.750-0.850	0.988	0.982-0.992
	Air (b=1500)	0.880	0.822-0.919	0.983	0.975-0.988
	Nodule (b=2000)	0.890	0.837-0.926	0.994	0.991-0.996
	Thyroid (b=2000)	0.839	0.816-0.889	0.811	0.772-0.887
	Air (b=2000)	0.922	0.884-0.947	0.995	0.992-0.996
Diagnostic performance	ADC (b=500)	0.837	0.806-0.857	0.892	0.808-0.894
	ADC (b=1000)	0.739	0.709-0.759	0.892	0.888-0.895
	ADC (b=1500)	0.941	0.913-0.906	0.889	0.883-0.892
	ADC (b=2000)	0.979	0.969-0.986	0.984	0.976-0.989
Sharpness	Nodule (b=500)	0.765	0.748-0.776	0.922	0.737-0.980
	Nodule (b=1000)	0.863	0.845-0.875	0.960	0.799-0.993
	Nodule (b=1500)	0.876	0.864-0.884	0.934	0.903-0.956
	Nodule (b=2000)	0.882	0.873-0.888	0.908	0.867-0.937
	Thyroid (b=500)	0.965	0.948-0.976	0.978	0.968-0.985
	Thyroid (b=1000)	0.746	0.720-0.764	0.915	0.874-0.943
	Thyroid (b=1500)	0.766	0.749-0.777	0.920	0.881-0.946
	Thyroid (b=2000)	0.846	0.750-0.877	0.919	0.880-0.946

ADC, apparent diffusion coefficient; CI, confidence interval; ICC, intraclass correlation coefficients.

### Evaluation of subjective image quality

**Figure 2:**
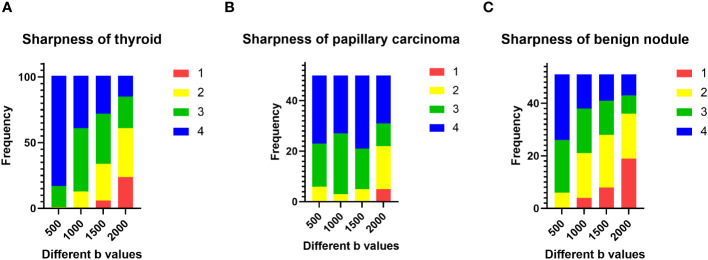
Evaluation of subjective image quality: **(A)** the sharpness of the thyroid decreased as the b value increased; **(B)** in the papillary carcinoma group, the sharpness of nodules was similar between b-values of 1000 s/mm^2^ and 1500 s/mm^2^; **(C)** in the benign nodule group, the sharpness of nodules was similar between b-values of 1000 s/mm^2^ and 1500 s/mm^2^.

The sharpness of thyroids decreased as the b value increased (**Figure 2A**). The multiple comparison results of sharpness are shown in **Table 2**. In both the papillary carcinoma group (*P* = 0.670) (**Figure 2B**) and benign nodule group (*P* = 0.185) (**Figure 2C**), the sharpness of nodules was similar between images with a b-value of 1000 s/mm^2^ and those with a b-value of 1500 s/mm^2^.

**Table 2:** The pairwise comparison of sharpness of nodules and thyroids with different b-values (×10^−3^ s/mm^2^) in DWI images.

	500-1000 (*P*)	500-1500 (*P*)	500-2000 (*P*)	1000-1500 (*P*)	1000-2000 (*P*)	1500-2000 (*P*)
Papillary carcinoma	0.877	0.786	0.002	0.670	0.004	< 0.001
Benign nodule	0.002	< 0.001	< 0.001	0.185	0.001	0.060
Thyroid	< 0.001	< 0.001	< 0.001	0.005	< 0.001	< 0.001

DWI, diffusion-weighted imaging.

### Evaluation of objective image quality

**Figure 3:**
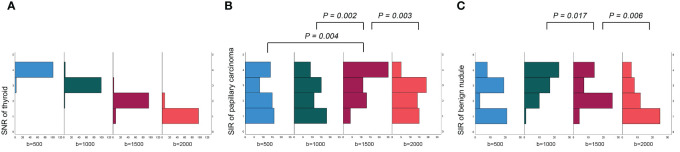
Evaluation of objective image quality: **(A)** the SNR of the thyroid decreased as the b value increased; **(B)** in the papillary carcinoma group, the SIR of nodules was statistically higher in DWI images with a b-value of 1500 s/mm^2^ than in DWI images with b-values of 500 s/mm^2^, 1000 s/mm^2^ and 2000 s/mm^2^; **(C)** in the benign nodule group, the SIR of nodules was statistically lower in DWI images with a b-value of 1500 s/mm^2^ than in DWI images with b-values of 1000 s/mm^2^ and 20000 s/mm^2^.

**Figure 4:**
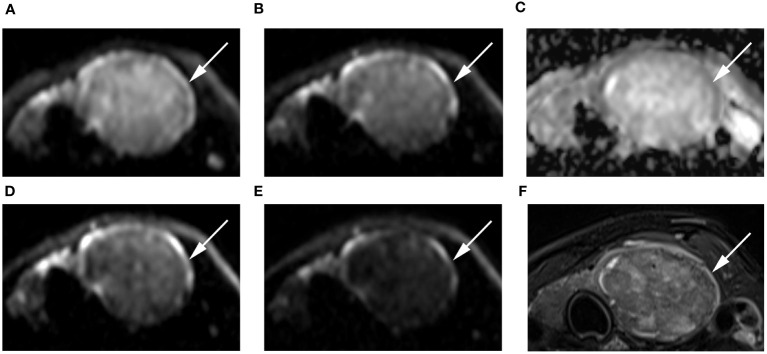
Images of a 55-year-old female with left lobe nodular goiters (arrow): DWI image with a b-value of 500 s/mm^2^ **(A)**, 1000 s/mm^2^ **(B)**, 1500 s/mm^2^ **(D)**, 2000 s/mm^2^ **(E)**; **(C)** ADC image with a b-value of 1500 s/mm^2^; **(F)** T2-weighted image. The SNR of the thyroid decreased as the b value increased. In the DWI image with a b-value of 1500 s/mm^2^, the SI of the nodule was significantly low relative to the other images.

**Figure 5:**
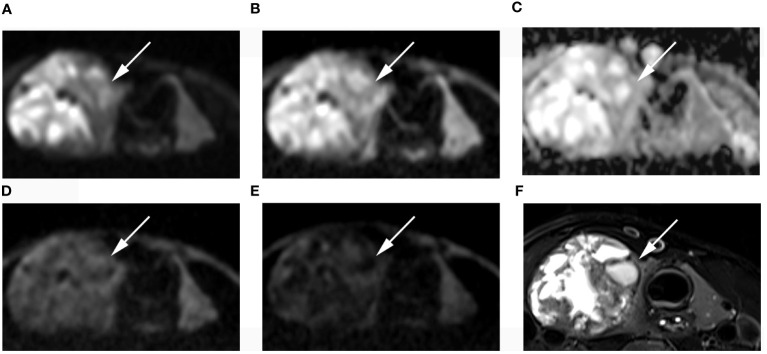
Images of a 49-year-old female with right lobe follicular adenoma (arrow): DWI image with a b-value of 500 s/mm^2^ **(A)**, 1000 s/mm^2^ **(B)**, 1500 s/mm^2^ **(D)**, 2000 s/mm^2^ **(E)**; **(C)** ADC image with a b-value of 1500 s/mm^2^; **(F)** T2-weighted image. The SNR of thyroid decreased as the b value increased. In the DWI image with a b-value of 1500 s/mm^2^, the SI of the nodule was significantly low relative to the other images.

**Figure 6:**
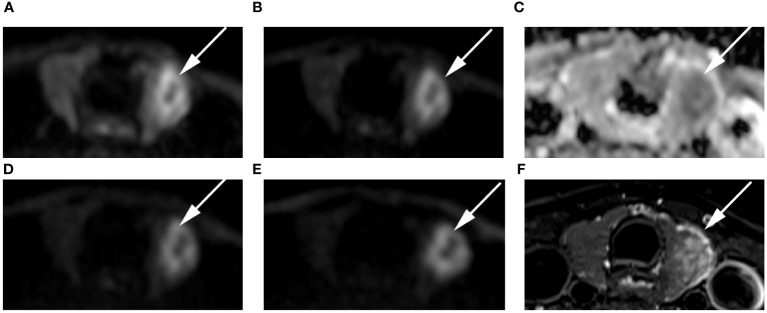
Images of a 54-year-old female with left lobe papillary carcinoma (arrow): DWI image with a b-value of 500 s/mm^2^ **(A)**, 1000 s/mm^2^ **(B)**, 1500 s/mm^2^ **(D)**, 2000 s/mm^2^ **(E)**; **(C)** ADC image with a b-value of 1500 s/mm^2^; **(F)** T2-weighted image. The SNR of the thyroid decreased as the b value increased. In the DWI image with a b-value of 1500 s/mm^2^, the SI of the nodule was significantly high relative to other out images.

**Figure 7:**
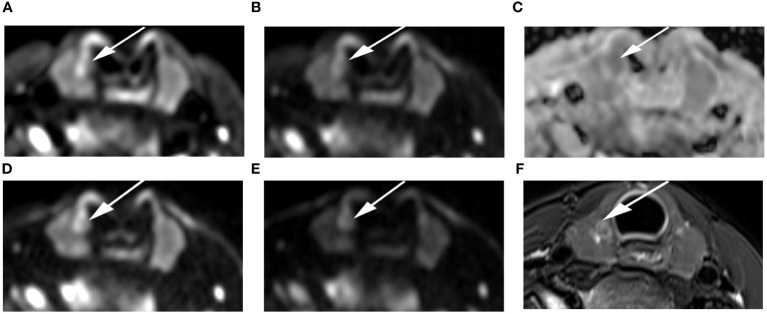
Images of a 24-year-old female with right lobe micropapillary carcinoma (arrow): DWI image with a b-value of 500 s/mm^2^ **(A)**, 1000 s/mm^2^ **(B)**, 1500 s/mm^2^ **(D)**, 2000 s/mm^2^ **(E)**; **(C)** ADC image with a b-value of 1500 s/mm^2^; **(F)** T2-weighted image. The SNR of the thyroid decreased as the b value increased. In the DWI image with a b-value of 1500 s/mm^2^, the SI of the nodule was significantly high relative to the other images.

The SNR_thyroid_ decreased as the b value increased (**Figure 3A**). In the papillary carcinoma group (**Figure 3B**), the SIR_nodule_ was statistically higher in DWI images with a b-value of 1500 s/mm^2^ than in DWI images with b-values of 500 s/mm^2^ (*P*= 0.004), 1000 s/mm^2^ (*P* = 0.002), and 2000 s/mm^2^ (*P* = 0.003). In the benign nodule group (**Figure 3C**), the SIR_nodule_ was statistically lower in DWI images with a b-value of 1500 s/mm^2^ than in DWI images with b-values of 1000 s/mm^2^ (*P* = 0.017) and 2000 s/mm^2^ (*P* = 0.006). The multiple comparison results of nodule sharpness are shown in **Table 3**. When the b-values were 1500 s/mm^2^ (*P* = 0.008) and 2000 s/mm^2^ (*P* = 0.009), the SIR_nodule_ significantly differed between the papillary carcinoma group and the benign nodule group (**Table 4**). In DWI images with a b-value of 1500 s/mm^2^, the difference in SI between malignant and benign nodules was visible to the naked eye (**Figures 4**–**7**).

**Table 3:** The pairwise comparison of SIR_nodule_ and SNR_thyroid_ with different b-values (×10^−3^ s/mm^2^) in DWI images.

	500-1000 (*P*)	500-1500 (*P*)	500-2000 (*P*)	1000-1500 (*P*)	1000-2000 (*P*)	1500-2000 (*P*)
SIR_papillary carcinoma_	0.816	0.004	0.877	0.002	0.938	0.003
SIR_benign nodule_	< 0.001	0.192	0.145	0.017	< 0.001	0.006
SNR_thyroid_	< 0.001	< 0.001	< 0.001	< 0.001	< 0.001	< 0.001

DWI, diffusion-weighted imaging; SIR, signal intensity ratio; SNR, signal-to-noise ratio.

**Table 4:** Comparison of the SIR_nodule_ values of papillary carcinomas and benign nodules with different b-values (×10^−3^ s/mm^2^) in DWI images.

	500	1000	1500	2000
Papillary carcinoma	0.61 ± 0.46	0.65 ± 0.80	1.05 ± 0.56	0.80 ± 0.65
Benign nodule	0.68 ± 0.58	0.65 ± 0.86	0.15 ± 0.43	0.10 ± 0.43
*P*	0.809	0.416	0.008	0.009

DWI, diffusion-weighted imaging; SIR, signal intensity ratio.

### Diagnostic performance evaluation

**Figure 8:**
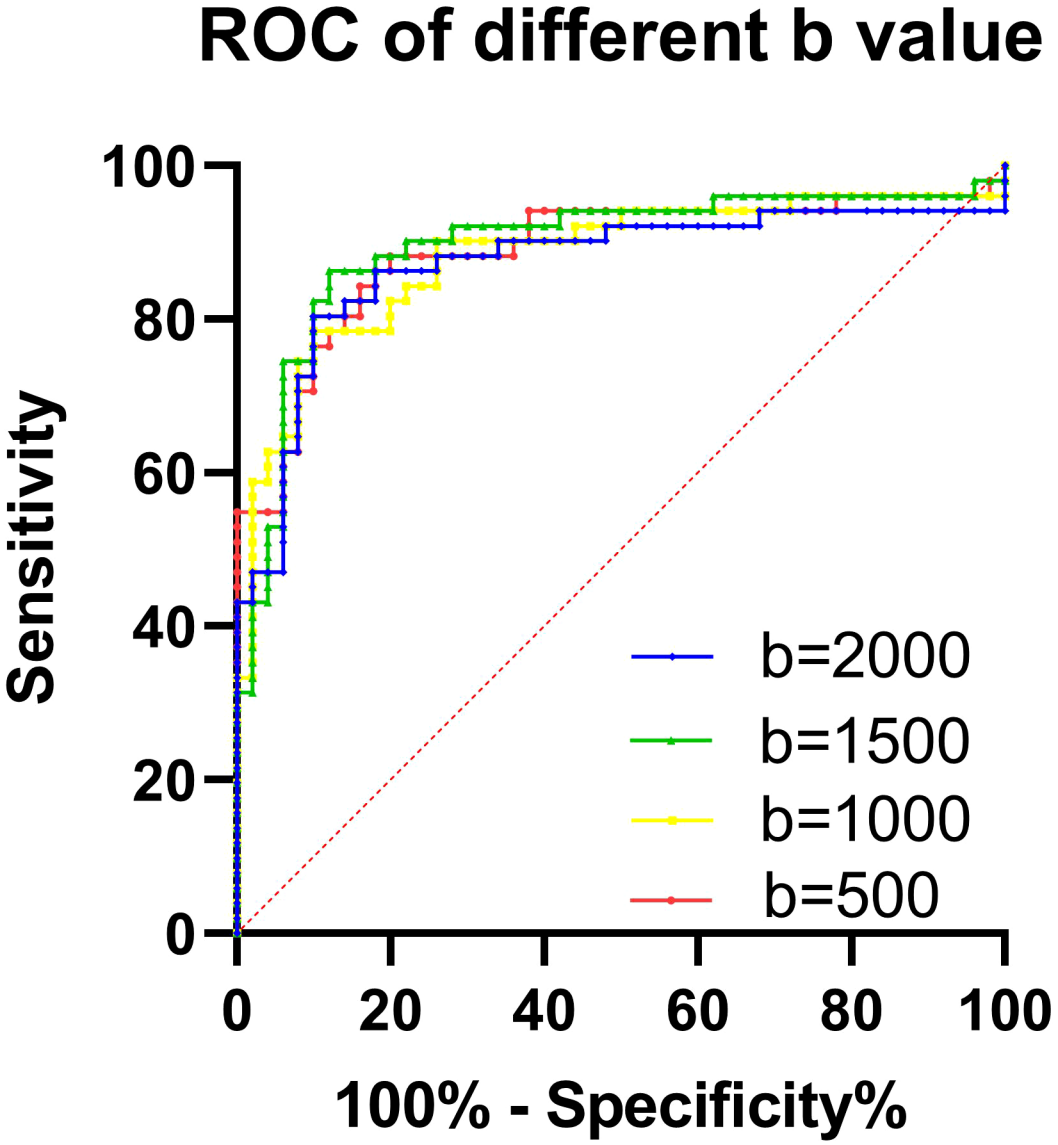
The ROC curve of the ADC with different b-values for predicting a papillary carcinoma.

Quantitative ADC measurements under different b-values all differed significantly between malignant and benign thyroid nodules (*P* < 0.05). The mean ADCs of malignant and benign thyroid nodules are shown in **Table 5**. When b = 500 s/mm^2^, the ADC had an AUC of 0.888, a sensitivity of 84.31%, and a specificity of 84.37%. When b = 1000 s/mm^2^, the ADC had an AUC of 0.881, a sensitivity of 78.34%, and a specificity of 89.97%. When b = 1500 s/mm^2^, the ADC had an AUC of 0.896, a sensitivity of 86.27%, and a specificity of 90.57%. When b = 2000 s/mm^2^, the ADC had an AUC of 0.871, a sensitivity of 80.39%, and a specificity of 89.99% (**Table 6**, **Figure 8**). The DeLong test showed comparable diagnostic effectiveness among the different b-value groups except for between b-values of 2000 s/mm^2^ and 1500 s/mm^2^, with a b-value of 2000 s/mm^2^ showing lower effectiveness (**Table 7**).

**Table 5:** Comparison of the mean ADC values of papillary carcinomas and benign nodules with different b-values (×10^−3^ s/mm^2^) in ADC images.

	500	1000	1500	2000
Papillary carcinoma (×10^−3^ mm^2^/s)	1.24 ± 0.30	1.03 ± 0.23	0.96 ± 0.24	0.79 ± 0.16
Benign nodule (×10^−3^ mm^2^/s)	2.10 ± 0.55	1.75 ± 0.46	1.46 ± 0.40	1.15 ± 0.31
*P*	0.003	< 0.001	< 0.001	< 0.001

ADC, apparent diffusion coefficient.

**Table 6:** Diagnostic performance of the ADC in differentiating between thyroid papillary carcinoma and thyroid benign nodules with different b-values (×10^−3^ s/mm^2^).

b value	500	1000	1500	2000
AUC (95% CI)	0.888 (0.819-0.957)	0.881 (0.810-0.952)	0.896 (0.828-0.964)	0.871 (0.794-0.947)
Sensitivity (95% CI)	84.31% (71.99%-91.83%)	78.43% (65.37%-87.51%)	86.27% (74.28%-93.19%)	80.39% (67.54%-88.98%)
Specificity (95% CI)	84.37% (71.49%-91.66%)	89.97% (78.64%-95.65%)	90.57% (76.20%-94.38%)	89.99% (78.64%-95.65%)
Cutoff ADC (×10^−3^ s/mm^2^)	1.47	1.28	1.16	0.93

ADC, apparent diffusion coefficient; AUC, area under the curve; CI, confidence interval.

**Table 7:** DeLong results for the ADC in predicting thyroid papillary carcinoma with different b-values (×10^−3^ s/mm^2^).

	500-1000	500-1500	500-2000	1000-1500	1000-2000	1500-2000
*P*	0.891	0.546	0.399	0.337	0.486	0.006

ADC, apparent diffusion coefficient.

## Discussion

DWI is an important method to differentiate malignant from benign lesions, but the most appropriate b-values for such differentiation are unknown because different tumors within different organs or tissues may have different sensitivities and specificities to different b-values. The purpose of this study was to explore the best b-value in ZOOMit-DWI to differential diagnosis. The results suggest that 1500 s/mm^2^ was a suitable b value for differentiating thyroid papillary carcinomas and thyroid benign nodules, which yielded better diagnostic performance and image quality. Thyroid papillary carcinomas and thyroid benign nodules can be differentiated by comparing signals from thyroid nodules on DWI images.

About the evaluation of subjective image quality, the sharpness of the thyroid decreased as the b value increased. The b-value is an important factor in DWI. On low-b-value images, the diffusion characteristic of tissues has only a small impact. Higher-b-value images are noisier and much darker (a low signal-to-noise ratio) and have the disadvantage of requiring considerable time for acquisition (16). Images with high b-values are of great significance in detection of benign and malignant nodules (17). In this study, when the b-values were 1500 s/mm^2^ and 2000 s/mm^2^, the SIR_nodule_ significantly differed between the papillary carcinoma and benign nodule. Because at higher b-values, tissues with high water molecule path lengths tend to lose signal rapidly (18). In addition, the b value plays a crucial role in ADC measurements. The ADC values are derived by DWI data to a monoexponential model using 2 b-values reflects tumor cellularity and thus the properties of diffusion restriction in tissue (19). ADC values are affected by both blood perfusion and extracellular space (20). Both in the papillary carcinoma and benign nodule, the ADC values decreased as the b value increased. The signal-to-noise ratio decreases as the b value increases (21).

In papillary carcinomas, the SIR_nodule_ was higher in DWI images with a b-value of 1500 s/mm^2^ than in DWI images with b-values of 500, 1000, 2000 s/mm^2^. In benign nodules, the SIR_nodule_ was lower in DWI images with a b-value of 1500 s/mm^2^ than in DWI images with b-values of 1000 and 2000 s/mm^2^. When the b-value was 1500 s/mm^2^, the SIR_nodule_ significantly differed between thyroid papillary carcinomas and thyroid benign nodules, suggesting that the SI difference was visible to the naked eye when the b-value was 1500 s/mm^2^. In addition, the ROC analysis with the ADC showed better classification results for b=1500 s/mm^2^ compared to the remaining b-values. Therefore, 1500 s/mm^2^ was the optimal b value for differentiating thyroid papillary carcinomas and thyroid benign nodules.

The sharpness of nodules and thyroids decreased as the b value increased. However, the sharpness of nodules was similar between images with a b-value of 1000 mm^2^/s and those with a b-value of 1500 s/mm^2^, suggesting that 1500 mm^2^/s maintains good image quality, which is inconsistent with results from a past study, where b values ranged from 200 s/mm^2^ to 800 s/mm^2^ on 1.5 T MRI, and worse image quality was noted at high b values (8). This discrepancy may be due to the ZOOMit technique, which improves image quality compared to conventional single-shot echo planar imaging.

In this study, thyroid imaging was performed with a 3.0 T MR scanner. In past literature, authors highlight the advantages of DWI on strong magnetic fields machines, which can accurately measure the ADC values (22). A 16­channel surface coil can provide better image signal-to-noise ratio performance and resolution for the thyroid, which ensures image quality. Past studies have shown that ZOOMit-DWI improves image quality compared with conventional DWI of the prostate, orbit and gallbladder (23–25), demonstrating that ZOOMit-DWI is a good tool to observe small organs. Due to the short scanning time, the relative homogeneity of signal excitation is increased, and image blurring and distortion are decreased (12). In this study, excellent image quality performance was achieved, which also benefited from ZOOMit-DWI. Good imaging quality is useful for manual ROI definition, which directly affects measurement results.

In this study, ADC maps were computed based on DWI images acquired with different b values and reflect the discrimination of thyroid papillary carcinoma from benign thyroid nodules. All past studies share one point of view: the diffusion of water molecules is restricted in malignant tumors, which lead to ADC values decrease, and the difference in the ADC values is valuable to make the differential diagnosis between benign and malignant tumors (26). This study also verified this view. Past studies demonstrated that the ADC values of malignant nodules was obviously lower than benign nodules in thyroid (6), which is similar to the results in this study, where the ADC value of thyroid papillary carcinomas was significantly lower than that of benign nodules regardless of whether the b-value was 500, 1000, 1500, or 2000 s/mm^2^. Papillary carcinomas have some microscopic features, which include psammoma bodies, foci of squamous metaplasia, lymphoid infiltration of the tumor stroma, and a peculiar appearance of tumor cell nuclei. The microscopic features lead to increased cellularity and reduced extracellular space (27). In addition, fibrosis also hampers the diffusion of water molecules to varying degrees (28).

This study has a few limitations. First, different field strengths were not compared. Second, only four b-values were compared in this study. In the future, we will narrow the b-value interval for verification. Finally, the ADC was measured by Siemens Healthcare equipment. Further research is needed to determine whether the cutoff value is appropriate for other MRI machines and third-party postprocessing platforms.

In conclusion, 1500 s/mm^2^ was a suitable b-value to differentiate benign and malignant thyroid nodules in ZOOMit-DWI images, which had better image quality, and a signal difference was visible to the naked eye.

## Data availability statement

The original contributions presented in the study are included in the article/supplementary material. Further inquiries can be directed to the corresponding author.

## Ethics statement

The studies involving humans were approved by Chongqing University Cancer Hospital institutional review board. The studies were conducted in accordance with the local legislation and institutional requirements. The participants provided their written informed consent to participate in this study. Written informed consent was obtained from the individual(s) for the publication of any potentially identifiable images or data included in this article.

## Author contributions

JW contributed to the conception and design of the study, data analysis and writing of the manuscript. LJ and DL contributed to performing the experiments and writing and revising the manuscript. JC, YT contributed to the data collection. JZ contributed to the data analysis and interpretation of the data. JZ is the guarantor of this study and approved the version to be submitted. All authors accept responsibility for the integrity of the data and the accuracy of the data analysis. All authors contributed to the article and approved the submitted version.
